# Effects of External Abdominal Pressure Support on Dynamic Balance: A Randomized Crossover Study

**DOI:** 10.3390/sports11110217

**Published:** 2023-11-07

**Authors:** Yuki Nakai, Takara Kijimuta, Yasufumi Takeshita, Ryoji Kiyama, Sota Araki, Takasuke Miyazaki, Masayuki Kawada

**Affiliations:** 1 Department of Mechanical Systems Engineering, Faculty of Engineering, Daiichi Institute of Technology, 1-10-2 Kokubuchuo, Kirishima 899-4395, Japanlog.a.d.e.3.2.1@gmail.com (Y.T.); 2 Department of Physical Therapy, School of Health Sciences, Faculty of Medicine, Kagoshima University, 8-35-1 Sakuragaoka, Kagoshima 890-8544, Japan; kiyama@health.nop.kagoshima-u.ac.jp (R.K.); kawada@health.nop.kagoshima-u.ac.jp (M.K.); 3 Course of Physical Therapist, Department of Rehabilitation, Faculty of Health Sciences, Tohoku Fukushi University, 1-8-1 Kunimi Aoba-ku, Sendai 981-8522, Japan; s-araki@tfu.ac.jp; 4 Department of Orthopaedic Surgery, Graduate School of Medical and Dental Sciences, Kagoshima University, 8-35-1 Sakuragaoka, Kagoshima 890-8544, Japan; takasuke0803@yahoo.co.jp

**Keywords:** balance, intra-abdominal pressure, performance, support belt, trunk

## Abstract

Abdominal pressure is vital in protecting the lumbar spine and controlling postural balance. Dynamic balance is associated with movement stability, adaptation to load, and reduced injury risk. Although trunk stability has been examined using belts and braces, the effects of external abdominal pressure support (APS) on balance control remain unknown. In this study, we aimed to determine the effects of external APS on dynamic balance. Overall, 31 young adults participated in this randomized crossover study. External APS was provided using a device that could be pressurized and decompressed by inflating a cuff belt wrapped around the trunk. The modified Star Excursion Balance Test was performed under external APS and non-APS conditions. The maximum anterior, posterolateral, and posteromedial values normalized to the spinal malleolar distance and their respective composite values were compared between the two conditions with and without APS. Posterolateral, posteromedial, and composite values were significantly higher in the APS condition than in the non-APS condition (*p* < 0.001). The external APS was effective in immediately improving dynamic balance. Furthermore, APS was effective in dynamic balance control as it improved stability during anterior trunk tilt, which displaces the center of gravity forward.

## 1. Introduction

All movements and forces of the body are linked through the myofascia surrounding the trunk, pelvic area, and lower extremities [1,2]. These structures interact and act as stabilizers that play an important role in maximizing performance [1]. In particular, effective mobilization of the trunk muscles is associated with optimal generation of muscle strength and precise control of hip motion through the lumbar spine and pelvis [3,4]. Conversely, trunk muscle weakness may lead to poor physical and balance performance [5]. Among the trunk muscles, the roles of the abdominal muscles are well known [6]. Abdominal bracing maneuvers that increase intra-abdominal pressure (IAP) by voluntary isometric contraction of the abdominal muscles are effective in increasing spinal stability [7,8,9]. Increasing abdominal pressure (AP) is important for generating muscle strength during lifting movements [10]. Increased AP also occurs during exertion and execution of lower extremity muscle groups such as in walking [11], jumping [12], and deadlift [10,13] movements. This phenomenon enables smooth execution of the main lower limb movement by stiffening the trunk prior to the movement [12]. The important factor in exerting lower limb muscle group strength is that an increase in AP may improve trunk stiffness. Therefore, when AP is externally supported, it may be associated with performance maximization of the trunk to lower extremity muscle groups. However, to the best of our knowledge, lumbar braces and belts have limited effects on AP [14]. These devices focus on tightening the lumbopelvic region and providing support to the lower back and do not add direct pressure manipulation from the abdomen to the AP. Additionally, consensus regarding the effectiveness of lumbar braces and belts is limited [15,16,17].

Strength, endurance, agility, speed, or other physical ability tests are used as surrogate measures of functional movement and athletic performance to examine the importance of trunk stability or lumbopelvic control [18,19,20]. The Star Excursion Balance Test (SEBT) is a dynamic balance test that is suitable for physically active individuals and does not require special equipment [21]. The SEBT predicts the risk of lower extremity injury [22,23,24] and identifies dynamic balance impairments in patients with lower extremity diseases [21,22,25,26]. Reliable dynamic balance tests are used to determine the effectiveness of training programs in healthy participants and patients with lower extremity diseases [21,27]. Although some studies have shown that foot and knee muscle strength and mobility predict dynamic postural stability, the relationship between the trunk and dynamic postural stability remains unclear [28]. Abdominal muscles are predictably activated earlier and exhibit greater amplitude in response to the direction of limb movement [29,30,31]. Regulating abdominal pressure can stabilize the spine independent of the direction of movement and also counteract moments imposed on the trunk [32]. Increased abdominal pressure improves spinal stability against off-balance perturbations [33,34]. Moreover, trunk and lower extremity perturbations when performing the SEBT may be influenced by the presence or absence of external abdominal pressure support (APS).

To our knowledge, no studies have investigated the effects of external APS on trunk stability during the SEBT. Therefore, we aimed to investigate the immediate effects of adding external APS during the SEBT on dynamic balance. The hypothesis was that the external APS would positively affect dynamic balance. This could be valid for external APS in rehabilitation, labor tasks, or various lifting activities. This study may provide insights into the future development of new lumbar orthoses and belts.

## 2. Materials and Methods

### 2.1. Participants

This study’s sample size was calculated based on reports on the effectiveness of lumbar stabilization exercises with a hollowing strategy for trunk flexor muscle strength [35]. A power analysis conducted using G* Power 3.1.9 (University of Kiel, Kiel, Germany) with a d of 0.53, α of 0.05, and power of 0.80, revealed that at least 30 participants were required for this study. Recruitment was conducted from February 2023 to April 2023 through announcements to students on the social network services of one engineering university and one healthcare professional school in Kirishima, Japan. The population consisted of approximately 240 students (85% male) in one grade at the university and 160 students (40% male) in one grade at the school, all aged between 18 and 22. In total, 34 students who were active and willing to voluntarily participate were included. This study was conducted in the order in which the schedules were matched. Inclusion criteria were the following: no trunk or lower extremity injury within the past 6 months, no trunk or lower extremity surgery in the past 2 years, no trunk or lower extremity pain, and no history of neurological disease. In addition, they had to have had previous experience in competitive sports. After excluding 3 persons with no previous competitive sports experience, a total of 31 young adults (19 male and 12 female participants; age, 20.0 ± 0.9 years; height, 166.5 ± 8.6 cm; body mass, 57.9 ± 8.1 kg) were enrolled.

### 2.2. Ethics

This study was approved by the Ethics Committee of Daiichi Institute of Technology (R4-003) and conducted in accordance with the principles of the Declaration of Helsinki [36]. The study was explained orally and in writing and written informed consent was obtained from all participants.

### 2.3. Study Design and Procedures

This was a randomized crossover study in which measurements with and without APS were performed on two separate days. All 31 participants were evaluated under both conditions on separate days with a washout period of at least 48 h [37]. The order was randomized using a random number table. Prior to the measurements, the participants performed a 5 min walk as a warm-up, 1 round trip up and down 50 stairs, and 5 repetitions of trunk flexion, extension, right and left rotation, and right and left lateral flexion exercises [38,39]. Three abdominal bracing repetitions were performed as a warm-up for the trunk muscles, with arms crossed in front of the chest in an upright position. A modified version of the SEBT (mSEBT) was used to measure dynamic balance [21]. The immediate effects of the presence or absence of APS were tested.

### 2.4. Measurement Methods

Body mass and height were measured prior to the study. Body mass was measured using a digital scale (MC-780MA; Tanita^®^, Tokyo, Japan), barefoot, with light clothing and accessories removed. Body height was measured using a height scale (seca213; seca^®^, Chiba, Japan), with the participants barefoot and in an upright posture. Measurements were taken to the nearest ±0.1 kg and ±0.1 cm, respectively.

**Figure 1:**
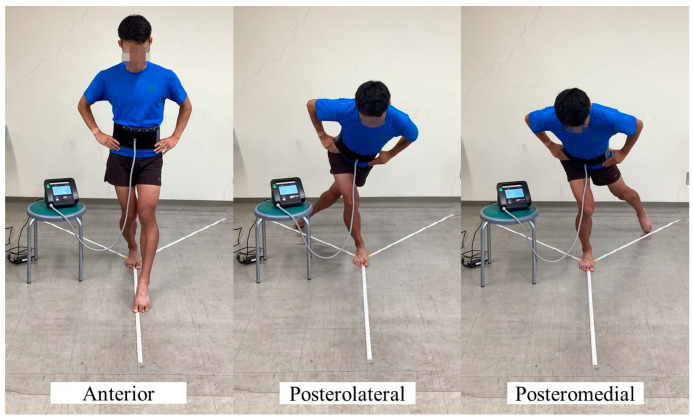
The mSEBT in the abdominal pressure measurement support setting. mSEBT, modified Star Excursion Balance Test.

In the SEBT, the participants maintained their balance on one leg while extending the opposite leg maximally in eight directions. A farther reach indicated better dynamic postural control. The mSEBT uses only three directions (anterior, posterolateral, and posteromedial) and has been validated for a shorter time on the task [21,23,25]. Therefore, the mSEBT was used in this study (Figure 1). In the questionnaire, the leg with which the participant could normally kick the ball harder was used as the dominant leg, and that leg was used as the static leg during the mSEBT [40]. The mSEBT was performed after four practice trials, followed by a 1 min break and three measurement trials [27,41,42], and the maximum value was adopted. A participant failed to complete the test and was retested if he/she (1) put weight on the reaching leg; (2) lost balance and could not return the extended leg to the starting position; (3) dropped their hands off their waist; (4) could not keep the standing leg in the same place; or (5) could not keep the forefoot or heel of the standing leg on the floor [21]. The maximum values in each of the three directions of the mSEBT and the means of their sum (composite) were normalized and expressed as percentages based on the spinal malleolar distance for comparison [43].

**Figure 2:**
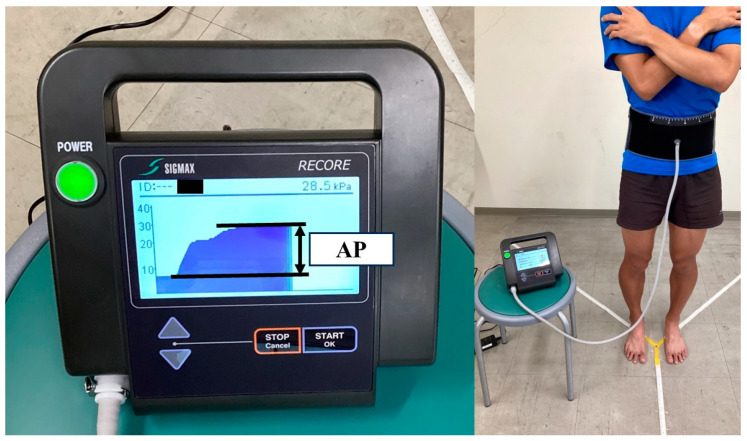
A participant wearing the abdominal trunk muscle strength-measuring device measuring their maximal abdominal pressure. AP, abdominal pressure.

External APS was provided using an abdominal trunk muscle strength-measuring device (RECORE^®^; Sigmax, Tokyo, Japan), which can be pressurized and decompressed by wrapping a 15 cm-wide cuff belt around the trunk and inflating it using air [44]. Initially, the abdominal pressure of all participants was measured three times while they were in the standing position, and the maximum value was defined as the maximum abdominal pressure (Figure 2). A biomechanical model simulation of the spine and its muscle structure was used to set and analyze a base pressure of 5 kPa corresponding to various movements [45]. This corresponded to approximately 30% of the mean maximum abdominal pressure among male participants in this study. As a pressure of 4 kPa corresponds to approximately 30% of the mean maximum abdominal pressure in female adults, the APS conditions during the mSEBT were set to 5 and 4 kPa for male and female participants, respectively. The non-APS condition was defined as simple wrapping of the cuff belt (0 kPa). The center of the cuff belt was 2.5 cm below the umbilicus, targeting the lower abdomen. The cuff belt was applied before the mSEBT trial, and the external APS was adjusted to the set value at the time of resting expiration, just before the commencement of the test. The application and setting of the APS system were performed by a physical therapist with sufficient experience.

### 2.5. Statistical Analysis

The reproducibility of the mSEBT measurements with and without APS was determined by calculating the intraclass correlation coefficient (ICC) (1, 3) using data from 13 young adults who had been assessed three times, each by one examiner prior to the study. The ICC of the mSEBT with the three-directional APS was 0.929–0.968 (0.824–0.989), whereas the ICC without APS was 0.946–0.981 (0.866–0.994). External abdominal pressure fluctuations during the mSEBT were also identified. The increase in abdominal pressure was confirmed by the difference between the maximum value of abdominal pressure during posterolateral implementation, which was assumed to cause trunk flexion, and the set value at the beginning of the measurement. The maximum values of three trials were used. The increases in the abdominal pressure were 4.92 ± 2.48 and 0.28 ± 0.19 with and without APS starting from set values of 5 and 0 kPa, respectively.

Data for each mSEBT item are presented as means and standard deviations. The data distribution for each item was determined to follow a normal distribution using the Shapiro–Wilk test. The effects of the presence or absence of APS on the mSEBT were compared using a paired *t*-test. SPSS version 28.0 (IBM Corp., Armonk, NY, USA) was used for all statistical analyses, and the significance level was set at *p* < 0.05. The effect size, d, calculated for comparison corresponded to the following criteria: trivial (<0.200), small (0.200–0.500), medium (0.500–0.800), and large (>0.800) [46].

## 3. Results

**Figure 3:**
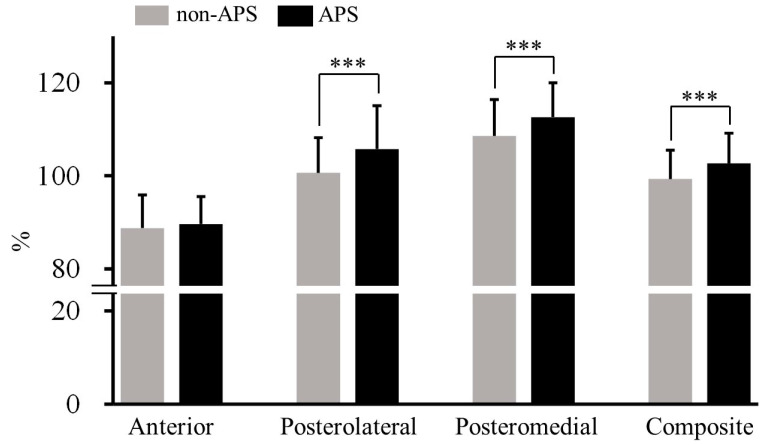
Comparison of each mSEBT score with and without APS. *** *p* < 0.001. APS, abdominal pressure support; mSEBT, modified Star Excursion Balance Test.

The participants’ characteristics are presented in Table 1. When comparing the mSEBT results with and without APS, no significant differences were present in the anterior direction (non-APS, 88.8 ± 6.0%; APS, 89.6 ± 7.2%; d = 0.19, trivial; p = 0.290). The values of the posterolateral (non-APS, 100.6 ± 9.4%; APS, 105.7 ± 7.7%; d = 0.74, medium), posteromedial (non-APS, 108.6 ± 7.5%; APS, 112.6 ± 7.9%; d = 0.70, medium), and composite of the directions (non-APS, 99.3 ± 6.6%; APS, 102.7 ± 6.3%; d = 0.73, medium) were significantly higher in the APS than in the non-APS settings (*p* < 0.001) (Figure 3).

## 4. Discussion

In this study, we examined the immediate effects of external APS on dynamic balance using the mSEBT. APS improved posterolateral performance by 5.1%, posteromedial performance by 3.7%, and composite performance by 3.4%. These results suggest that external APS may be effective for supporting dynamic balance in young adults. However, factors such as lower extremity muscle strength, range of motion, intrinsic receptivity, and neuromuscular control were not evaluated, so their associations with these factors are not known.

Lumbar belts and braces support spinal stability by tightening the abdominal wall and increasing the IAP, which reduces the load on the spine [47,48]. Simultaneous contraction of trunk flexors and extensors increases the IAP, and the longitudinal moments acting on the pelvis and diaphragm reportedly increase trunk stiffness and reduce intervertebral pressure [45,47,49]. This mechanism of action is similar to that of abdominal bracing, which induces higher activation of deep abdominal muscles, such as the internal oblique muscles [50]. These trunk-stabilizing muscles are constantly activated before the activation of the limbs [51]. This is thought to assist force or power generation of the limbs during kinetic chain activity [52,53]. A strong and stable trunk and its rapid activation are potentially the foundation for limb force generation and achieving improved sports performance [1]. A 4 kPa increase in the IAP results in a 25% improvement in spinal stability [54]. Furthermore, trunk muscle-strengthening exercises enhance spinal stability and postural control in patients with lower back pain (LBP) [55]. We previously confirmed that APS increased the AP by an average of 4.9 kPa during movements in the posterolateral direction. In contrast, in the non-APS setting, in which the cuff belt was simply wrapped around the trunk, the AP increased by 0.28 kPa during movements in the same posterolateral direction. This suggests that APS may have improved spinal stability during the mSEBT.

Spinal somatosensory acuity has been reported to influence changes in the trunk muscles and postural control [16,56]. Lumbar somatosensory dysfunction has been observed in patients with LBP [57]. However, the lumbar belt may partially provide somatosensory information to the lower back of patients with LBP [58]. APS may not only tighten, but the air pressure supporting the lower abdomen may also directly stimulate somatosensory sensation and promote AP activation. Considering these factors, a significant improvement in dynamic balance may have been observed with APS during movements in the posterolateral direction. The same reason could explain the same trunk flexion movements during the movements in the posteromedial direction. Thus, in the current study, APS would have provided immediate support for trunk stability in young adults during movements in the posterolateral and posteromedial directions and would have shown an overall improvement in dynamic balance scores.

In this study, when assessing the effectiveness of APS, we observed improvements in scores for the posterolateral and posteromedial directions, whereas no significant differences in scores for the anterior direction were present. Posture is controlled by activating the transversus abdominis muscle under the influence of different body positions and loading styles [32,59]. Wearing a belt or brace increases the hip flexion angle during lifting movements, in which the center of gravity shifts forward [60], and increases muscle activity in the internal oblique and rectus abdominis muscles [61,62]. In addition, an appropriate increase in IAP enhances the maximum voluntary contractile torque of hip extension [63]. Compared with movements in the posterolateral and posteromedial directions, which exhibit an anterior trunk tilt, movements in the anterior direction did not require a lumbar extension moment and required less lumbar stability. Lumbar shear load is strongly influenced by the lumbar flexion angle [64]. Thus, APS provided adequate support and improved dynamic balance when lumbar spine stability was needed during posterolateral and posteromedial movements when the hip flexion angle increased and the center of gravity moved forward due to the anterior tilt of the trunk. In the anterior direction, on the other hand, the need for lumbar spine stability was low because the lumbar spine was not flexed, so the APS was not needed as much and was not effective. Nevertheless, APS was beneficial during posterolateral and posteromedial movements, during which the center of gravity shifted forward, improving stability during anterior trunk tilt, which suggests its effectiveness for dynamic balance control and postural maintenance. Personalized lumbar orthoses that change shape using actuators or bands are currently available [65,66]. APS features focus on abdominal pressure and its effect on postural changes, not just tightness. As this design provides support using pneumatic pressure, changes can be easily personalized according to the individual’s movement; furthermore, the absence of pillars reduces concerns related to problems caused by contact. The results of this study may lead to the development of an autonomously modifiable lumbar brace with built-in software that allows air pressure to vary according to posture.

This study had some limitations. First, the participants were healthy young adults with a history of competitive sports; older adults or patients with comorbidities were not included. Therefore, these results may not be generalizable to other groups. Second, dynamic balance tasks require consideration of lower extremity muscle strength, range of motion, intrinsic receptivity, and neuromuscular control [23,26,67,68]. As these factors were not assessed in the present study, their relevance remains unclear. Third, it remains unclear which APS strategies changed because of the lack of an objective assessment of joint parameters and muscle activity during movement. Finally, the equivalence of IAP and AP has not been rigorously investigated. Further studies on the sustained effects in various populations are required to thoroughly understand the mechanisms underlying the improvement of dynamic balance.

## 5. Conclusions

This study examined the immediate effects of external APS on dynamic balance during the mSEBT for healthy young adults. External APS resulted in immediate improvements in dynamic balance, primarily during anterior trunk tilt. These results suggest that external APS may be an effective way to support dynamic balance during forward bending movements such as picking up objects and lifting.

## Figures and Tables

**Table 1:** Participants’ characteristics.

Variable	Total	Male	Female
Participants (number)	31	19	12
Age (years)	20.0 ± 0.9	20.4 ± 0.9	19.4 ± 0.6
Height (cm)	166.5 ± 8.5	172.0 ± 4.9	157.7 ± 4.7
Body mass (kg)	57.9 ± 8.0	62.0 ± 6.4	51.5 ± 5.6
Competition history (years)	5.1 ± 1.3	5.1 ± 1.3	5.1 ± 1.3
Maximal abdominal pressure (kPa)	15.1 ± 4.5	17.3 ± 4.1	11.7 ± 2.5
Spinal malleolar distance (cm)	83.0 ± 4.5	84.8 ± 4.0	80.2 ± 3.7

The values are presented as means ± standard deviations.

## Data Availability

Not applicable.
